# Mass fatality preparedness among medical examiners/coroners in the United States: a cross-sectional study

**DOI:** 10.1186/1471-2458-14-1275

**Published:** 2014-12-15

**Authors:** Robyn RM Gershon, Mark G Orr, Qi Zhi, Jacqueline A Merrill, Daniel Y Chen, Halley EM Riley, Martin F Sherman

**Affiliations:** Department of Epidemiology and Biostatistics and Institute for Health Policy Studies, School of Medicine, University of California, San Francisco, San Francisco, CA 94118 USA; Virginia Bioinformatics Institute, Virginia Tech, 900 N Glebe Rd, Arlington, VA 22203 USA; School of Nursing and Department of Biomedical Informatics, Columbia University, 617 W. 168th Street, Georgian, 226, New York, NY 10034 USA; Association of Schools and Programs of Public Health (ASPPH), Atlanta, GA 30333 USA; Department of Psychology, Loyola University Maryland, Baltimore, MD 21210 USA

**Keywords:** Disasters, Mass fatality incident, Preparedness, Medical examiners, Coroners, Death care, CBRNE, Ability and willingness

## Abstract

**Background:**

In the United States (US), Medical Examiners and Coroners (ME/Cs) have the legal authority for the management of mass fatality incidents (MFI). Yet, preparedness and operational capabilities in this sector remain largely unknown. The purpose of this study was twofold; first, to identify appropriate measures of preparedness, and second, to assess preparedness levels and factors significantly associated with preparedness.

**Methods:**

Three separate checklists were developed to measure different aspects of preparedness: MFI Plan Elements, Operational Capabilities, and Pre-existing Resource Networks. Using a cross-sectional study design, data on these and other variables of interest were collected in 2014 from a national convenience sample of ME/C using an internet-based, anonymous survey. Preparedness levels were determined and compared across Federal Regions and in relation to the number of Presidential Disaster Declarations, also by Federal Region. Bivariate logistic and multivariable models estimated the associations between organizational characteristics and relative preparedness.

**Results:**

A large proportion (42%) of respondents reported that less than 25 additional fatalities over a 48-hour period would exceed their response capacities. The preparedness constructs measured three related, yet distinct, aspects of preparedness, with scores highly variable and generally suboptimal. Median scores for the three preparedness measures also varied across Federal Regions and as compared to the number of Presidential Declared Disasters, also by Federal Region. Capacity was especially limited for activating missing persons call centers, launching public communications, especially via social media, and identifying temporary interment sites. The provision of staff training was the only factor studied that was significantly (positively) associated (*p* < .05) with all three preparedness measures. Although ME/Cs ranked local partners, such as Offices of Emergency Management, first responders, and funeral homes, as the most important sources of assistance, a sizeable proportion (72%) expected federal assistance.

**Conclusions:**

The three measures of MFI preparedness allowed for a broad and comprehensive assessment of preparedness. In the future, these measures can serve as useful benchmarks or criteria for assessing ME/Cs preparedness. The study findings suggest multiple opportunities for improvement, including the development and implementation of national strategies to ensure uniform standards for MFI management across all jurisdictions.

**Electronic supplementary material:**

The online version of this article (doi:10.1186/1471-2458-14-1275) contains supplementary material, which is available to authorized users.

## Background

History is replete with examples of disasters resulting in catastrophic numbers of fatalities. Just in the last decade, a wide range of natural and anthropogenic global events resulted in extremely high mortality rates in the affected communities. Examples include the 2001 World Trade Center attacks (~3,000 deaths), the 2003 Western Europe heat wave (~35,000 deaths), the 2004 South Asian tsunami (~220,000-230,000 deaths), the 2005 Kashmir earthquake (~75,000 deaths), 2008 Sichuan, China earthquake (~87,000), the 2009-10 H1N1 pandemic (~20,000 deaths), the 2010 Haiti earthquake (~200,000 deaths), the 2011 Japan mega-disaster (~22,000 deaths), and the recent 2014 West Africa Ebola virus disease epidemic (~6,400 deaths) [1–3]. In some cases, these massive fatality incidents completely overwhelmed local and even national capacity to respond appropriately, resulting in both acute and long-term adverse impacts on survivors and communities [4–6]. Although they are difficult to prepare for, the well-documented association between ineffective mass fatality management and adverse impacts on survivors and communities is leading to an increased focus on management of mass fatality incidents; the United States (US), in particular, has recognized this as a high priority of disaster planning [7].

Both large-scale fatality disasters, as well as smaller scale incidents with multiple fatalities are referred to as “*mass fatality incidents*” (MFI). Generally, we use the term MFI to describe situations in which the *resultant number of deaths exceeds the local jurisdiction*’*s ability* to *respond effectively*. More recently, another term, referred to as “*Complex Fatality Management*” (CFM), is being used in recognition of the fact that local capacity can be overwhelmed by *even a single fatality* if the incident involves hazardous chemical, biological, radiological, nuclear or explosive (CBRNE) agents. (Personal communication, Cynthia Galvin, 2014).

In the US, the medico-legal authority for decedents is typically under the purview of Medical Examiners or Coroners (ME/Cs). In most jurisdictions (usually county-level), ME/Cs are responsible for the investigation and management of deaths resulting from homicide, suicide, and accidents as well as deaths resulting from incidents that may present a threat to public health [8]. Offices of ME/C, spread across over 1000 jurisdictions in the US, can range from very small offices, essentially manned by one-person, to much larger and robust offices, with more than one hundred employees [9]. Guidance on MFI management is generally provided in a state’s mass fatality response plan, which many states have, usually as an annex to the state’s disaster plan [10]. The local ME/C may also have office-specific MFI plans. Although having a written plan is an important first step, ME/Cs must also have adequate operational capabilities to execute the plan. Pre-existing relationships with response partners, including governmental agencies, local businesses, and voluntary organizations, can be vital to ensuring ME/Cs offices’ response capacity. These partners may supply additional staff, space, supplies, or other forms of support.

In response to MFI, the ME/C must execute or oversee an array of operational tasks. Some of these include: securing and preserving human remains at a disaster site; recovering human remains; developing and implementing public communication messages; credentialing and managing volunteer staff; mobilizing missing persons call centers; performing all morgue operations, including ante-mortem and postmortem data collection for victim identification (Victim Identification Program); transporting, storing and securing temporary interment of remains; and releasing human remains for final disposition [11–15]. Furthermore, if CBRNE agents have contaminated the scene and/or human remains, then the ME/C must also take special precautions to ensure the safety of their staff and community. ME/Cs must also consider the impact of their investigative actions with respect to religious rituals or faith traditions. ME/Cs should also be cognizant of and make preparations for reducing adverse mental health impacts of the MFI response on staff and volunteers.

In the US, a mechanism exists for local jurisdictions to request disaster assistance, described in the National Response Framework (NRF), the nation’s guide for responding to all-hazards disasters [7]. Under this framework, the Department of Health and Human Services (DHHS) is responsible for coordinating MFI needs [16], and upon request, one of the important assets that DHHS can deploy are the services of Disaster Mortuary Operational Response Teams (DMORT). These highly qualified and skilled teams can bring supplies and expertise to MFI to help augment local capacity. However, even when DMORT teams are deployed, ME/Cs are responsible for the initial MFI management and for requesting this aid [17]. Because of the complexity of mass fatality management operations, there is a growing concern among ME/Cs regarding preparedness for MFI and their ability to manage MFI competently, especially for an incident that involves CBRNE [14].

These types of concerns were formally raised nearly a decade ago when a panel of national experts was convened by the US Northern Command, which provides command and control of the Department of Defense homeland defense efforts. The panel, referred to as the Joint Task Force Civil Support Mass Fatality Working Group (“Working Group”), was charged with examining the available data to determine the response capabilities and preparedness of the US mass fatality infrastructure for managing high fatality events, such as pandemics [12]. The Working Group identified several key elements of preparedness for the management of mass deaths and acknowledged significant knowledge gaps regarding the extent to which these elements had been adopted. The Working Group concluded that knowledge regarding the nation’s ability to manage a mass fatality event was limited. Since that time, many different initiatives have been undertaken to help ensure the nation’s readiness to manage MFI, and most currently, in 2014, a high level Mass Fatality Management Executive Steering Committee was formed to help provide effective guidance on MFI preparedness and management (Personal communication, Cynthia Galvin, 2014).

Since ME/Cs arguably have the most critical role to play in the US mass fatality infrastructure, and because preparedness and response capabilities for this group have not, to our knowledge, been previously assessed across the nation, the purpose of this study was as follows: first, to develop criteria for measuring preparedness in this sector; second, to assess subjective and objective preparedness and operational capabilities; and third, to identify organizational characteristics and other correlates of preparedness. This information is valuable in developing mass fatality management benchmarks as well as serving as an indicator for assessing actual preparedness. The ultimate goal of this study was to improve nation-wide MFI capabilities.

## Methods

### Study design and participants

This cross-sectional study was conducted over a six-week period in 2014. A self-administered, anonymous survey was made available on a SSL-secured site using a web-based tool [18]. Participants were adult professionals in the Medical Examiner/Coroner field and were recruited through newsletters, websites, and mass emails with the assistance and support of professional ME/Cs organizations. All study procedures involving human participants had prior review and approval of the University of California, San Francisco (UCSF) Human Research Protection Program, Committee on Human Research (CHR) (approval number 12-09425) and Columbia University Human Research Protection Office Institutional Review Boards (approval number AAAL0206), and informed electronically signed consent was obtained from each participant enrolled in this study.

### Questionnaire development and design

The preparedness measures were developed through an exhaustive four-part process involving the assessment of existing materials and review by experts in mass fatality management and emergency preparedness and response. As a starting point for the measures, national documents, such as the 2014 National Response Framework [7] and the National Response Plan (NRP) [19], and in particular, the NRF Emergency Support Function #8, the Public Health and Medical Services Annex [16], were carefully reviewed. A core functional area under ESF #8, is “mass fatality management, victim identification, and decontamination of remains,” for which the Department of Health and Human Services has primary and coordinating responsibility. The delineation of the supplemental role of the federal government was necessary for comparison and clarity of the ME/C responsibilities all jurisdictional level [5]. The second step in developing the measures was to conduct an environmental scan of existing state annexes or mass fatality plans as well as other available documents on mass fatality planning and response from the National Association of Medical Examiners (NAME) and the International Association of Coroners and Medical Examiners, the two leading professional associations for ME/C [15, 20–26]. Additionally, at this stage, we also reviewed toolkits and checklists developed by several state mass fatality planners, and we also reviewed the British Columbia Coroners Service (BCCS) Mass Fatality Response Plan [27–29]. These key documents provided the reference point for developing the preparedness measures, which were conceptualized as consisting of three domains: (1) Mass Fatality Plan Elements; (2) Mass Fatality Response Operational Capabilities, and (3) Pre-existing Resource Networks. In the third step, draft items for each of the measures of preparedness were then prepared and submitted for review and assessment to more than a dozen nationally recognized subject experts and key informants. These included the lead authors of highly developed state and regional plans, members of the national mass fatality planning steering committee, leadership of national professional ME/C organizations, and emergency management and mass fatality planning leaders. Our goal was to obtain consensus on the content validity of these new measures. In the fourth and final step, 11 representatives of the target population (ME/Cs) were asked to pre-test the computerized version of these measures and other elements of the questionnaire so that we could assess face validity of the measures as well as readability and length of time for completion. A copy of the ME/C questionnaire and codebook are appended in an Additional files 1 and 2, and all other documents related to the development of the measures may be obtained by contacting the corresponding author. The study questionnaire was written in English and prepared at a 13.5 grade reading level for ease of completion (length of time to complete ranged from 12-20 minutes) [30]. Most items used “yes”, “no”, “don’t know” response categories or discreet categorical responses. The three preparedness measures used a simple checklist box to indicate a positive (“yes”) response. The questionnaire included items that addressed organizational characteristics, MFI preparedness measures, and staff ability and willingness to report to duty, which is conceptualized as an important outcome related to preparedness [31–34].

### Measures

#### Organizational characteristics

Seven items were used to characterize the respondent’s organization, including office type (Medical Examiner vs Coroner or other), location (zip code), population size of jurisdiction served, whether urban or rural setting, number of employees, number of additional fatalities in excess of normal case load within a 48-hour period that would exceed capacity, and MFI experience in the past 5 years.

#### Mass Fatality Incident (MFI) preparedness

MFI Preparedness was assessed by three new categorical (nominal) measures: (a) Mass Fatality Plan Elements, operationalized as a 19-item checklist derived primarily from sample state mass fatality plans and the National Association of Medical Examiners (NAME) MFI management procedures [35]; (b) Mass Fatality Response Operational Capabilities, measured with a 21-item checklist developed with input from field experts and sample state, and national guides [14, 15, 20, 27, 28]; and (c) Pre-existing Resource Networks, measured with a new 12-item checklist of jurisdictional and community resource partners.

#### Additional MFI planning

Seven items helped to further characterize MFI planning, including: (a) an office-specific MFI plan and frequency of updating the plan; (b) the plan’s compliance with the National Incident Management System (NIMS) and with the Federal Emergency Management Agency (FEMA) Comprehensive Preparedness Guide (CPG) [36, 37]; (c) the jurisdictional role for ME/C during MFI; (d) interoperability and mutual aide agreements; (e) written policies on public communication during MFI; (f) use of social media during MFI; and (g) provision of mental health/spiritual counseling to staff and/or volunteers during and after MFI.

#### Staff willingness and ability to report to duty, with and without contamination with CBRNE agents

This was assessed using a two-part item based on some of our earlier studies on ability (i.e., availability) and willingness of staff to report to duty [31–34]. For these items, respondents were asked to indicate the proportion of staff that they thought would be willing and/or able to report to duty during MFI, with or without CBRNE as follows: (a) proportion of staff who they thought would be willing to report in their roles during a mass fatality incident; (b) proportion of staff who they thought would be willing to report in their roles during a mass fatality incident that involved CBRNE contaminants; (c) proportion of staff who they thought would be able to report in their roles during a mass fatality incident; (d) proportion of staff who they thought would be able to report in their roles during a mass fatality incident that involved CBRNE contaminants. Additional items were included on the preparation of a staff roster and staff pre-event planning in order to determine the availability of staff during MFI.

#### Training of staff and participation in drills

Three items addressed training, including training of staff on the office’s mass fatality plan, training of staff on MFI involving CBRNE agents, and training of the office through participation in jurisdictional drills.

#### Self-reported workplace and jurisdictional preparedness

Measured by two items on respondents’ perceptions of the preparedness levels of (a) their office, and (b) their local jurisdiction.

#### Resource needs

One final item asked respondents to indicate from a list of seven resources (plus an additional open ended “other” response category) the resources they thought they needed to help improve their office’s MFI preparedness and response. The list included such items as “more training”, “more planning activities”, “more funding for preparation activities”, “more interagency agreements”, etc.

As noted, a copy of the study questionnaire and codebook are appended.

#### Data analysis

After checks for internal reliability and validity of responses and other data editing procedures were completed, an array of descriptive statistics and graphical techniques (e.g., frequencies, histograms, measures of central tendency and dispersion) were performed to characterize the distribution of variables and to determine if there were any outliers. This strategy provided familiarity with the data and allowed us to determine if the data met assumptions required by the intended statistical testing procedures. All analyses were conducted using R (version 3.1.0, Auckland, New Zealand) [38].

The main outcome (criterion) variables were the three measures of MFI preparedness. To determine the relationship between the three measures, Pearson's product-moment correlation coefficient, *r*, was used to measure the degree of linear association between each of the variables, with results ranging from *r* = .49 - .64, indicating generally moderate to strong correlations. After careful examination of the frequency distribution of the three outcome variables, each outcome measure was dichotomized into two categories (scores below the median = 0; equal or above the median = 1). This was appropriate given that the data were bimodally distributed (to include zeros) and because these were categorical and not continuous variables. The dichotomization also allowed for visual depictions of the preparedness measures averaged across Federal Regions. This also facilitated visual comparison with the number of Presidential Disaster Declarations, 2001-2014, also averaged across Federal Region [39]. The maps as depicted were created using ArcGIS 10.2.2 for Desktop (Redlands, CA) [40].

To explore the organizational factors associated with preparedness, we first performed chi - squared statistics and estimated odds ratios and their confidence intervals using bivariate logistic regression analysis between each predictor and outcome measure in order to provide insight into the non-adjusted relations between predictors and outcomes. The next stage in our analysis involved logistic multivariable analysis to determine the unique relationship between the outcome variables (preparedness measures) and each predictor variable when considering all variables simultaneously. Linear regression was not used as the preparedness variables were, as noted, categorical and not continuous variables. For all regression analyses, the level of significance was set at 5%. Results are presented as estimated odds ratio (*OR*) and their 95% confidence interval (95% CI).

## Results

### Organizational characteristics

**Figure 1:**
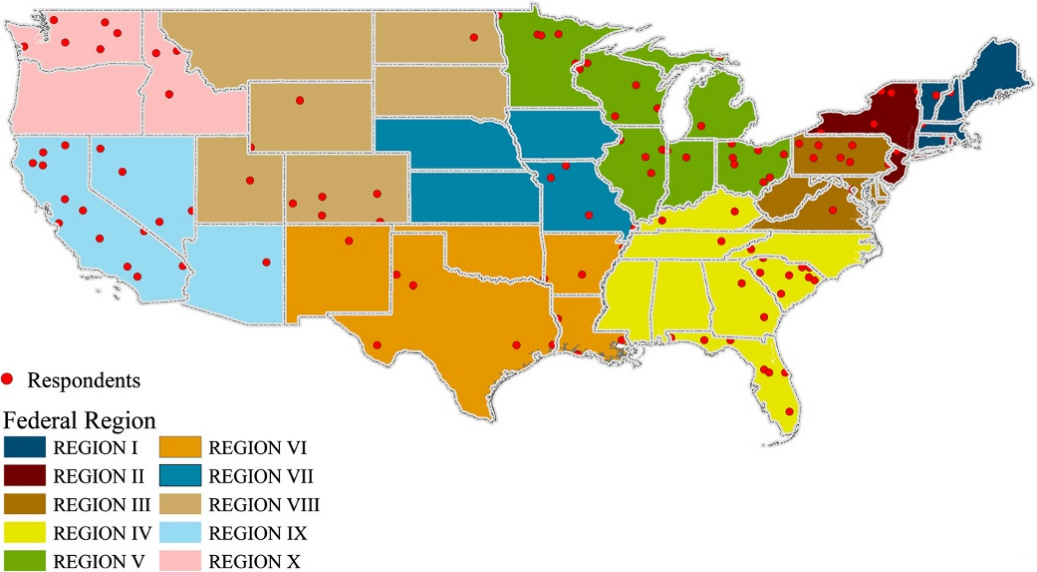
**Distribution of ME/** **C respondents in US.** Federal Regions were colored differently and the states within each region were separated using black lines. State and zip code data were used from the questionnaire to determine the location of each ME/C respondent (red dots).

A total of 122 completed questionnaires were collected. The actual response rate cannot be calculated, as this was a convenience sample. However, the number of ME/C in the US is approximately 900-1,000 [9], based on the most recent (2004) available data. The mapped distribution of responses by US states and territories is shown in Figure 1. The sample represented each of the 10 Federal Regions and 37 of the 56 states and territories [41]. Most of the respondents were either Medical Examiners (48%) or Coroners (44%), with the remainder indicating another role, including Forensic Pathologist (2%), Sheriff (2%), Justice of the Peace (1%), and “other” (3%). The majority of respondents (49%) indicated that their office served large jurisdictions (≥500 K people), followed by 25% serving 100 K-499.9 K, 14% serving 50 K-99.9 K, 8% serving 25 K-49.9 K, and 4% serving small jurisdictions with populations below 25 K. On average, most agencies (52%) employed ≤10 full time employees, with only 9% indicating ≥ 100 employees.

### MFI preparedness and additional MFI planning

#### MFI plan elements

Frequencies for the MFI Plan checklist items are shown in Table 1. A large majority of respondents (95%) reported that their office had a written mass fatality plan, but only 9% of the sample reported having all 19 items on the MFI Plan Elements Checklist included in their plan; on average, respondents reported having 68% of the plan elements in their own plans (checklist mean = 12 [*SD* = 6.2], median = 13, min = 0, max = 19). The most common plan items reportedly in place were: morgue services; human remains recovery; and command and control. The least frequently noted plan items included: job action sheets for the various positions in the plan; funding reimbursement policies; and availability of staff respite areas.

**Table 1:** **Mass fatality plan frequencies (N = 114)**

	***n*** (%) ^a^
Morgue services	101 (88.6)
Human remains recovery	96 (84.2)
Command and control	94 (82.5)
Security and preservation of the remains	92 (80.7)
Incident notification and plan activation	85 (74.6)
Authorities	81 (71.1)
Family assistance	81 (71.1)
Concept of operations	80 (70.2)
Security and preservation of the disaster site	79 (69.3)
Application and scope	75 (65.8)
Continuity of operations plan	73 (64.0)
Religious/cultural considerations (e.g., disaster emotional and spiritual care)	67 (58.8)
Mass fatality information systems	65 (57.0)
Vital records system	62 (54.4)
Credentialing, managing and documenting disaster personnel, including volunteers	57 (50.0)
Assumptions	55 (48.3)
Job action sheets for the various positions in the plan	49 (43.0)
Staff respite area	37 (32.5)
Funding reimbursement	33 (29.0)

^a^Data shown represent individuals who endorsed each item on the checklist.

Respondents reported that their local ME/C office’s plan was updated annually (32%), every two years (22%), or every five years (13%), with only 3% reporting that it was never updated; a sizeable proportion (30%) were unsure or unaware of how often the plan was updated. A large proportion (70%) indicated that their plan was compliant with NIMS [36]. However, only 40% reported that their plan was compliant with the FEMA CPG [37]. Engagement at the local level was adequate; a large percentage (77%) reported that their office had a seat at their local jurisdiction’s Emergency Operations Center during MFI, and an even greater proportion (88%) had a defined position during MFI. Most respondents (78%) had interoperability and mutual aide agreements in place. A large percentage (75%) of respondents reported having written policies that addressed public communications/public announcements during MFI, but only 29% had written policies related to the use of social media during MFI. Less than half (48%) of the respondents had plans in place that addressed the provision of mental health care or spiritual counseling for their staff and/or volunteers during or after MFI.

#### Operational capabilities

Only 9% of respondents reported all 21 Operational Capability Checklist items (mean = 10 [*SD* = 5.9], median = 11, min = 0, max = 21); on average, respondents reported having 52% of the operational capabilities in place. The most frequently reported operational capabilities for managing MFI included: refrigerated storage of remains, postmortem examination/morgue operations and transport of remains. The least frequently cited capabilities included: long term family management (i.e., memorial services), availability of temporary interment facilities, and communication system in place using social media. See Table 2 for a complete list of the frequencies for this checklist.

**Table 2:** **Operational capabilities frequencies** (**N** = **117**)

	***n*** (%) ^a^
Refrigerated storage of remains	94 (80.3)
Decedent recovery	89 (76.1)
Postmortem examination/morgue operations	89 (76.1)
Transport of remains	89 (76.1)
Decedent release/final disposition	82 (70.1)
Command and control for fatality management	81 (69.2)
Security and preservation of human remains	80 (68.4)
Ante-mortem data collection	74 (63.3)
Tracking system (i.e., victim identification Program) for recovered remains	69 (59.0)
Joint agency death investigation	67 (57.3)
Decedent manifest	59 (50.4)
Information technology/tracking	52 (44.4)
Morgue operations for contaminated (hazard material) human remains	50 (42.7)
Public messaging	46 (39.3)
Incident characterizations	42 (35.9)
Security and preservation of disaster site	42 (35.9)
Caring for or interring human remains in accordance to the religious ritual or requirements of most faith traditions	37 (31.6)
Missing persons call centers	28 (23.9)
Communication via social media	26 (22.2)
Temporary interment	21 (18.0)
Long term family management/memorial	10 (8.6)

^a^Data shown represent individuals who endorsed each item on the checklist.

#### Pre-existing resource networks

The frequencies for the items on the Pre-existing Resource Networks checklist are shown in Table 3. Only 8% of respondents reported having relationships with all 12 potential sources of resources needed to respond to MFI. The median checklist score for this measure was 8.5 (mean = 8 [*SD* = 3.3], min = 0, max = 12); on average, the sample had more than 70% of the potential pre-existing relationships in place. The most frequently reported pre-existing partnerships in place were with local organizations and agencies, such as local office of emergency management, local members of the death care sector (organizations affiliated with the funeral industry), and the local department of health. A substantial proportion (72%) also reported that they planned to rely on federal assets. Most respondents (75%) reported that their jurisdictional partners had their own mass fatality plan, but less than 30% had signed off on those plans. Typically, the response partners that the ME/Cs expected to assist them in MFI were the same ones that they, in turn, would plan to help.

**Table 3:** **Pre**-**existing resource networks frequencies** (**N** = **118**)

	***n*** (%) ^a^
Local Office of Emergency Management/Civil Defense	99 (83.9)
Local funeral homes, cemeteries, crematories	97 (82.2)
Local first response organizations	95 (80.5)
Local/State Department of Health	92 (78.0)
State Office of Emergency Management/Civil Defense	88 (74.6)
Federal assets	85 (72.0)
Voluntary organizations	79 (67.0)
Local health care organizations	78 (66.1)
Other nearby Coroner/Sheriff’s office/Justice of the Peace	74 (62.7)
Other nearby office of medical examiner	65 (55.1)
Faith-based organizations	51 (43.2)
Disaster management vendors/contractors	47 (39.8)
Other	4 (3.4)

^a^Data shown represent individuals who endorsed each item on the checklist.

### Graphic comparison of the three preparedness measures

**Figure 2:**
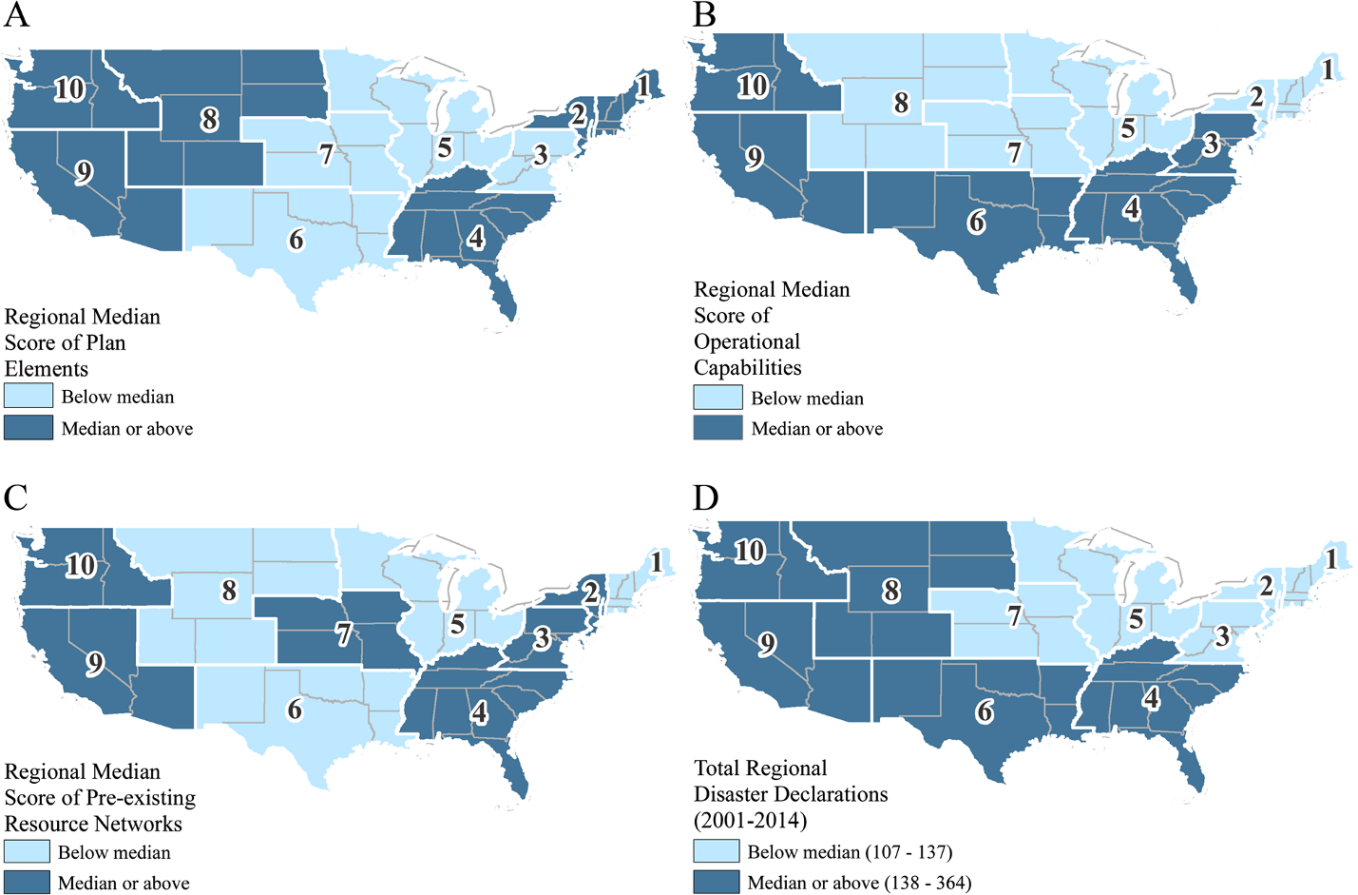
**Comparisons between preparedness measures scores and with respect to the Presidential Disaster Declarations. (A)** MFI Plan measure by Federal Region; **(B)** Operational Capabilities measure by Federal Region; **(C)** Pre-existing Resource Networks measure by Federal Region; and **(D)** Presidential Disaster Declarations (2001-2014) by Federal Region. Data for the disaster declarations map were available at the FEMA website [27] and median number of regional disaster declarations (N = 138) was used to categorize all 10 regions in map D. Maps A-C were created using questionnaire data. Scores for the preparedness measures and number of disaster declarations were categorized into two groups (below median and equal to or above median). The lighter blue represents regions with scores/number of disaster declarations below the median and the darker blue indicates regions with scores/number of disaster declarations equal to or above the median.

A graphic comparison of the three measures and total Federal Regional Presidential Disaster Declarations from 2001-2014, by Federal Region, is provided in Figure 2(A,B,C,D). As noted, we have stratified the preparedness scores for each of the three measures into two categories (below the median and equal or above the median) across each of the Federal Regions [41]. As can be seen, scores vary across the three measures, as shown in 2(A-C). For example, only Region 4, which includes Alabama, Florida, Georgia, Kentucky, Mississippi, North Carolina, South Carolina, and Tennessee; Region 9, which includes Arizona, California, Hawaii, Nevada, and the Pacific Islands; and Region 10, which includes Arkansas, Idaho, Oregon and Washington, had uniformly higher scores on all three preparedness measures.

Other findings of interest relate to visual comparisons with the map depicting the Presidential Disaster Declarations (2001-2014), by Federal Region. Between 2001 and 2014, there were 1,740 declarations made in the US [39]. The total number of Disaster Declarations by Federal Region has been categorized into two groups: < the median and ≥ the median number of reported declarations, *per* Federal Region) over the time period of 2001-2014. As can be seen in Figure 2(D), the levels of preparedness within each Federal Region are at the same level or higher than the median number of declared disasters in all regions except for Region 6 (Texas, New Mexico, Oklahoma, Arkansas, and Louisiana), and Region 8 (Utah, Colorado, Wyoming, Montana, South Dakota and North Dakota) [41].

### Prior mass fatality incident experience and the number of additional fatalities within a 48-hour period that would exceed capacity

A majority (82%) of respondents reported that their jurisdiction had *not* experienced MFI in the past 5 years. A sizeable proportion (42%) of the respondents reported that their capacity would be exceeded by the addition of 24 or less fatalities (over and beyond their normal case load) over a 48-hour time period. Additional fatalities that would exceed capacity responses were as follows: 25-50 additional fatalities (28%), 51-75 additional fatalities (10%); 76-100 additional fatalities (4%), and 13% of respondents reported that they had the capacity to manage an additional 100 fatalities or more in a 48-hour period.

### Staff willingness, ability to report to duty, with and without contamination with CBRNE agents

A large majority (83% and 72%) of respondents indicated that 80% or more of their staff would be *willing* and *able*, respectively, to report to duty during a mass fatality incident. However, substantially lower proportions (46% and 52%) reported that 80% or more of their staff would be *willing* and *able*, respectively, if MFI involved CBRNE agents. Nearly one quarter of the respondents reported that they expected *less than 20*% of their staff to report if CBRNE agents were involved in MFI. Furthermore, whereas staff rosters had generally been prepared to indicate staff availability, less than half of the respondents thought that staff had made pre-event plans (e.g., plans to address childcare and elder care responsibilities) that would ensure their availability to work during MFI. Results of chi-squared analyses indicated significant positive relations between training of staff and staff availability (*p* < .01) and between staff participation in drills and willingness to report to duty (*p* < .01), suggesting that training may help support adequate staffing.

### Training of staff and participation in drills

More than 70% of respondents indicated that their office provided training to staff on the mass fatality plan, and staff participation in drills with local partners was common (79%). However, CBRNE–related MFI training was much more limited; only 27% of the respondents reported that their office had ever provided this type of specialized training.

### Perceptions of office and jurisdictional preparedness

Less than half (43%) of the sample thought that their office was well prepared to manage MFI, 16% thought that they were not at all or slightly prepared, and the reminder (41%) thought they were moderately prepared. Similarly, more than half (55%) of the respondents thought their jurisdiction was well prepared, 10% thought that their jurisdiction was not at all or slightly prepared, and the rest (35%) thought they were moderately prepared.

### Resources needed to help improve MFI preparedness and response

Only 5 (4%) respondents indicated that their office did not need any additional resources to improve their preparedness. The resources most frequently needed included more training for staff, greater surge capacity, and more funding for mass fatality planning. Detailed results may be found in Table 4.

**Table 4:** **Resources needed to improve MFI preparedness and response** (**N** = **120**)

	***n*** (%) ^a^
Additional training of staff	89 (74.2)
Greater surge capacity (identification of additional staff, supplies, space)	78 (65.0)
Additional funding for mass fatality planning	77 (64.2)
Additional mass fatality planning activities	71 (59.2)
Additional drills with other response partners	70 (58.3)
Signed interagency agreements	51 (42.5)
Other (e.g., better communications, CBRNE trainings, better coordination, etc.)	19 (15.8)
A written mass fatality plan	15 (12.5)
ME/C office does not need any additional resources to be better prepared	5 (4.2)

^a^Data shown represent individuals who endorsed each item on the checklist.

### Relations between preparedness measures and predictor variables

As noted in Table 5, several factors were significantly associated with each of the measures of preparedness, but only one variable, *training of staff on the office*’*s MFI plan*, was significantly associated with all three. Training of staff was also the single most strongly associated variable with respect to the MFI Plan measure (*OR* = 5.44, 95% CI [2.10, 14.11]); i.e., ME/Cs who provided staff training on the mass fatality plan had a 5.44 greater odds of a higher score compared to those ME/C reporting no staff training. Similarly, staff training was also positively associated with the Operational Capabilities and Pre-existing Resource Networks measures (*OR* = 3.86, 95% CI [1.58, 9.46] and *OR* = 2.86, 95% CI [1.19, 6.84]), respectively. For the Operational Capabilities measure, the strongest association was noted for *maximum fatalities* that could be handled within 48 hours (*OR* = 5.65, 95% CI [2.52, 12.66]); ME/C reporting that they could handle 25 or more fatalities within 48 hours had a 5.65 greater odds of having a higher score on the Operational Capabilities measure than those who could handle 24 or less within 48 hours.

**Table 5:** **Bivariate regression analysis of preparedness measures and organizational**

	MFI plan elements ***N*** = 114		Operational capabilities ***N*** = 117		Pre-existing resource networks ***N*** = 118	
	Odds ratio 95% CI	***p-*** value	Odds ratio 95% CI	***p-*** value	Odds ratio 95% CI	***p-*** value
Workplace category (coroner is the reference category)	1.03	.934	1.03	9.34	0.34	.006**
	[0.42-2.17]		[0.49-2.17]		[0.16-0.74]	
Number of full time employees (6 or less is the reference category)	1.77	.129	2.52	0.16*	0.78	.500
	[0.85-3.72]		[1.19-5.34]		[0.37-1.62]	
Maximum fatalities that can be handled within 48 hrs (24 or less is the reference category)	2.25	.025*	5.65	< .001***	1.01	.970
	[1.06-4.78]		[2.52-12.66]		[0.48-2.12]	
Having experience of mass fatality incidents in the past 5 years (no is the reference category)	2.23	.135	3.54	.022*	1.12	.810
	[0.79-5.71]		[1.20-10.41]		[0.44-2.88]	
Training on mass fatality plan (no or not have a plan is the reference category)	5.44	< .001***	3.86	.003**	2.86	.018
	[2.10-14.11]		[1.58-9.46]		[1.19-6.84]	
Training on CBRNE (no is the reference category)	1.86	.155	2.17	.075	1.32	.510
	[0.79-4.38]		[0.92-5.12]		[0.58-3.02]	
Drills participation (no is the reference category)	4.68	.005**	3.83	.003**	1.75	.250
	[1.59-13.73]		[1.58-9.46]		[0.68-4.48]	
Having staff roster (no is the reference category)	1.83	.184	5.48	.001**	2.38	.061
	[0.75-4.47]		[1.99-15.14]		[0.96-5.87]	
Proportion of staff that are willing to report to duty during a regular mass fatality incident (70% or less staff willing is the reference category)	2.94	.042*	2.55	.068	2.25	.110
	[1.04-8.32]		[0.93-6.98]		[0.82-6.13]	
Proportion of staff that are willing to report to duty during a CBRNE involved mass fatality incident (70% or less staff willing is the reference category)	1.88	.177	1.87	1.27	1.10	.820
	[0.85-4.13]		[0.84-4.17]		[0.50-2.39]	
Proportion of staff that are able to report to duty during a mass fatality incident (70% or less staff able is the reference category)	1.53	.313	2.24	.058	1.28	.550
	[0.67-3.49]		[0.97-5.15]		[0.56-2.91]	
Proportion of staff that are able to report to duty during a CBRNE involved mass fatality incident (70% or less staff able is the reference category)	1.27	.552	1.15	.730	0.92	.830
	[0.58-2.76]		[0.52-2.52]		[0.42-2.00]	
Proportion of staff that have pre-event plan (70% or less staff able is the reference category)	1.15	.762	1.77	.224	2.34	.072
	[0.47-2.80]		[0.70-4.46]		[0.93-5.89]	
Self-reported workplace preparedness (Less prepared is the reference category)	3.40	.002**	5.27	< .001***	1.56	.250
	[1.55-7.45]		[2.31-12.00]		[0.74-3.28]	
Self-reported jurisdiction preparedness (Less prepared is the reference category)	2.35	.025*	2.69	.010*	1.35	.420
	[1.12-4.95]		[1.27-5.70]		[0.65-2.81]	
Serving urban area (less than 50,000 is the reference category)	2.14	.199	1.65	.386	0.55	.300
	[0.67-6.81]		[0.53-5.07]		[0.17-1.74]	
MFI plan elements (below median is the reference category)	-	-	6.71	< .001***	2.93	.004**
			[3.04-14.82]		[1.40-6.11]	
Operational capabilities (below median is the reference category)			-	-	2.22	0.31*
					[1.01-4.59]	
Pre-existing resource networks (below median is the reference category)					-	-

Note. All organizational characteristics and preparedness measures were coded into binary variables.

*p < 0.05. **p < 0.01. ***p < 0.001.

Prior experience managing MFI was significantly associated with higher scores on the Operational Capabilities checklist (*OR* = 3.54, 95% CI [1.20,10.41]). Self-reported perceptions of organizational preparedness were strongly (positively) associated with both the MFI Plan and the Operational Capabilities measures, as noted in Table 5, indicating that respondents had perceptions that reflected at least their own actual level of readiness as measured by these two preparedness measures, although we have no information on the actual readiness at the jurisdictional level.

Findings of interest include the general lack of a significant relation between preparedness measures and the size of jurisdiction served, although the Operational Capabilities measure was significantly (positively) associated with a larger workforce (>6 full time employees).

### Relations between preparedness measures and willingness and ability of staff to report to duty

Conceptually, willingness and ability of staff is seen as *resulting from* preparedness of the worksite. That is, the more prepared the workplace, the more willing and able the work force will be to report to duty during disaster events, including MFI. However, since these data are cross-sectional, directionality for any of these study variables cannot be ascertained. To assess the relationship between the willingness and ability variables and the three preparedness measures we included them, as shown in Table 5, in the bivariate analyses. The preparedness measures, for the most part, were not significantly associated with staff willingness and ability to report to duty during MFI, with or without contamination with CBRNE. One exception here was the moderate positive association between staff willingness to report to non-contaminated MFI and (higher) MFI Plan measure score (*OR* = 2.94, 95% CI [1.04, 8.32]).

### Results of the multivariate analysis

Most variables significant at the bivariate level were no longer significant at the multivariate level. Exceptions included the MFI Plan measure and Operational Capabilities measure, which remained significantly correlated to each other (OR = 3.44, 95% CI [1.27,9.32], p < .05); ME/Cs reporting highly developed plans were more than three times more likely to report a high level of organizational capabilities. Another significant result at both the bivariate and multivariate levels was that Coroners (as compared to Medical Examiners) were more likely to report a higher score on the Pre-Existing Resource Network measures (OR = 3.5, 95% CI {1.46,8.40, p < .01]. A borderline significant score was also noted between the MFI Plan measure and training on the MFI Plan.

## Discussion

Based on our results, the three new preparedness measures presented in this paper provide for a broad and inclusive perspective on mass fatality management preparedness. The MFI Plan measure serves as a road map for planning, the Operational Capabilities allows for an in-depth assessment of organizational resources and Pre-existing Resource Networks indicates the external supports available to the ME/Cs. These three measures, when combined, reflect a more accurate level of local ME/C preparedness to manage MFI. A fourth component, ability and willingness of staff can also be considered an important indicator of preparedness, but we conceptualize this as an *outcome* of preparedness (i.e., staff who perceive a more prepared workplace will be more willing to show up for work during times of disaster), rather than a *predictor* of preparedness. The nature of the willingness and preparedness relationship has been clearly demonstrated in real time during the Ebola virus outbreak, where well prepared biocontainment hospitals have reported a large increase in job applicants willing to work in these high containment facilities in contrast with hospitals treating cases or suspected cases without this degree of preparedness reporting job actions and strikes [42, 43].

The results of the bivariate analysis indicate the important relationship between high quality planning and training; since these data are cross sectional, the exact nature of the relationship for instance, between measures of preparedness and training, cannot be determined. However, it does seem likely that ME/Cs with high quality planning also are more likely to implement training programs based on their planning. For the most part, factors that were significant in the bivariate analyses were no longer significant in the multivariable analysis, probably due to collinearity. More complex study designs and the inclusion of other variables not studied here, such as the funding available for MFI planning at the jurisdictional level and MFI training and knowledge of the ME/C, are needed in order to better characterize the factors related to preparedness.

Our three-tiered approach to measuring preparedness contrasts with other models for systems-level preparedness, which have been suggested, such as the Ready, Willing, and Able framework developed by McCabe and colleagues [44]. Our approach incorporates many of their constructs, especially “Ability”, which is reflected in our operational capabilities measure. In our efforts to characterize preparedness, we examined other metrics considered by field experts to be essential components, such as resources available to the ME/C through pre-existing relationships and agreements- as these are vital to effective MFI management. Additionally, in line with systems-level preparedness models, our approach also acknowledges “willingness” as noted above as an important component of preparedness. Compared to other sectors we have studied with respect to willingness, the ME/C are reportedly very willing to report to duty [31, 32]. Still, and in consideration of the fact that ME/Cs and staff are under a legal mandate to report to duty, a large proportion of staff may fail to report-especially if CBRNE agents are involved. Importantly, even if staff are willing, their availability may be severely limited during an infectious disease outbreak causing staff illness; meeting surge capacity needs for staffing could then be highly problematic, as replacement staff most likely would not have the unique skill set required of ME/C and staff.

Our findings indicate variable levels of preparedness using three separate, yet related measures of preparedness. While improvements are indicated in all aspects of preparedness, special attention is needed to address operational capabilities since, on average, respondents had only about half of the items on the Operational Capabilities Checklist in place. Without these core capabilities, response will be limited.

With respect to the map of Presidential Disaster Declarations comparing the median scores of the three preparedness measures, while there are some differences, it is important to acknowledge that not all disasters result in high fatalities; a good example of this was Super Storm Sandy, which, while causing an estimated $50B in damages, resulted in relatively few direct deaths (*N* = 147) given the magnitude of the disaster [45].

Some notable findings in our study include the fact that preparedness levels did not differ based on type of office (i.e., Medical Examiner vs Coroner), jurisdictional characteristics (size of population and rural vs urban), or the number of staff. These findings indicate that preparedness may not be simply a function of organizational characteristics, but potentially influenced by some other factor(s). It was also notable and reassuring to find that there was a high degree of inter-organizational planning in place, as this strengthens individual ME/C capabilities. As noted, our results also point towards the need for special attention for certain aspects of preparedness, such as the use of social media to help communicate with the public, and mobilizing missing person’s hotlines. Social media is increasingly being used by the public during and in the immediate aftermath of disasters to help them connect to family, friends, resources and timely information [46, 47].

There was a gap noted in terms of providing mental health assistance to staff and volunteers and the provision of long-term family assistance, which may be particularly important in the wake of MFI [48]. There are a number of excellent information resources on the provision of mental health and spiritual care, such as the Interfaith Network of trained religious and lay leaders who are available for both planning and response purposes [49]. We noted that many respondents intended to call upon their local death care sector colleagues for instrumental support and they may also be able to play a role in terms of family assistance and staff respite centers.

Results from this study indicate that, in some jurisdictions, local ME/Cs are well equipped, staffed and prepared to respond to MFI, while in other jurisdictions, they might be overwhelmed by any additional fatalities beyond their typical case load. Complex situations involving CBRNE would challenge *almost all* ME/Cs. The recent outbreaks of infectious diseases such as Middle East Respiratory Syndrome (MERS), Ebola virus disease and recent influenza pandemics, as well as a number of industrial and transportation incidents involving hazardous chemicals underscore the need for preparedness for these types of incidents [3, 50]. ME/Cs must look to their local office of emergency management and their local health departments for advice on what types of training and preparedness activities are feasible and meaningful. At the very least, local ME/Cs should know the agency or persons to contact for expert advice if there they ever have to respond to a CBRNE incident.

The findings here are in agreement with both the first (2012) and the second (2013) National Preparedness Reports; these serve as the nation’s report card for documenting progress in building, sustaining, and delivering the 31 core capabilities as outlined in the National Preparedness Goal [13, 51, 52]. The 2012, 2013 and the most recent 2014 reports highlighted the need for improvements in mass fatality preparedness [13, 51, 53]. Planning for MFI has been historically subpar; FEMA reported that between 2006 and 2011, only 24 out of 56 states and territories invested Department of Homeland Security grant funds for fatality management activities, and fatality management services were rated as the *weakest* of all 31 core response mission capabilities [13]. Since we merged our findings and presented them by Federal Region, state level differences are not easily observed. However, at the regional level we can ascertain wide differences across the nation. These geographical differences may reflect the variable levels of investment in mass fatality planning across states. In the 2013 national review of state’s fatality management plans, it was noted that while most states had established fatality management plans, upon review some were inadequate or not actionable [13]. This observation is consistent with our findings at the local ME/C level, which showed that while individual ME/Cs reported the existence of plans, there was a lack of completeness, as assessed by our new measures. Furthermore, more than half of the states do not expect to be able to build additional capacity and therefore intend to rely on federal assets to close existing gaps [13]. Notable exceptions to this include outstanding progress made in certain jurisdictions including New York City, Harris County Texas, Florida, Alabama, Ohio and several others [13], which may serve as models for states struggling to develop adequate capacity. Increased efforts to improve MFI preparedness have also been made through the outreach provided to dozens of jurisdictions by the National Transportation Safety Board (NTSB), the formation of Regional Catastrophic Planning Teams, such as the New York, New Jersey, Connecticut and Pennsylvania team; the FBI establishment of the Scientific Working Group in Disaster Victim Identification and the availability of an integrated web-based Unified Victim Identification System, developed by the NYC Office of the Chief Medical Examiner, and training programs developed and hosted by the National Mass Fatalities Institute [13, 54–56].

### Recommendations

Although these data have important limitations, namely that they were obtained using convenience sampling and a cross-sectional design, these findings nevertheless represent the largest sample of ME/C to comprehensively report on MFI preparedness and response capabilities. Based on these results, a number of preliminary recommendations are made.

1. MFI Plan templates, tailored to local jurisdictional capabilities, should be made widely available through appropriate channels, including the national organizations representing the ME/C.
2. The Operational Capabilities Checklist that we have developed may also be a useful tool that could be made widely available. Dissemination of tools like this checklist may help local ME/Cs develop high quality planning. MFI plans, operational capabilities and resource sharing agreements should be reviewed by ME/Cs annually and updated as needed. The development and upkeep of plans should be a transparent process, and information should be distributed effectively throughout the organization. The fact that many respondents did not know how often their plans were updated indicates that this is not currently the reality for most ME/Cs. Contact information, including chain of command (local and state level) contacts should always be kept up-to-date.
3. Because training was associated with preparedness, it would be helpful to have web-based training made widely available to all ME/Cs to ensure that they are trained effectively using up-to-date curricula, and excellent resources for this, including programs addressing CBRNE, are available [21, 57–59]. Drills were also important. The local jurisdiction office of emergency management should take the lead in organizing MFI drills so that all responding actors can participate, including local death care industry businesses, local first responders, representatives of faith-based organizations, and others.
4. ME/C leadership should identify one or more experienced “advisors” within each Federal Region who can coach local ME/Cs through the most important first steps in the immediate aftermath of MFI and to help the local ME/C in forming the appropriate questions to ask of response agencies. Additionally, every ME/C must have ready access to experts knowledgeable on the management of fatalities that involve CBRNE agents, as very few ME/C have the necessary resources to safety and effectively manage incidents involving these hazardous agents.
5. Since it is clear that many local ME/Cs expect to be provided with resources at the federal level, a dialog between local, state and federal level responders must develop and be ongoing.
6. Funding is needed at national, regional, and local levels for MFI preparedness and for the development and implementation of mass fatality management best practices guidance.
7. Finally, study data should be reviewed by key informants in order to triangulate the findings and to ensure adequacy of these preliminary recommendations.

### Strengths and limitations

A major strength of this study is that this is, to our knowledge, the first study to develop MFI preparedness criteria to assess subjective preparedness, and the first to assess national levels and correlates of preparedness. The criteria were developed with the input of many respected ME/Cs in the field, which strengthens the measures and indicates a high level of professional interest in improving MFI response capabilities.

The three measures (a combined total of 52 items) could be made widely available for ME/Cs to rapidly conduct self-assessments, taking no more than 10 minutes to complete. The results might guide tailored quality improvement activities, such as inter-agency agreements with local response partners. The measures could also be used at local and regional drills to assess MFI preparedness at the state and Federal Region level. Other countries could adapt these measures to meet their own national requirements. The measures could also be used as a post-assessment of MFI response; in the aftermath of MFI, local ME/C could conduct a self-evaluation of their response. Data from these measures also provide support for on-going national efforts to improve the quality and effectiveness of MFI management capabilities.

There are also several potential study limitations. First, with a cross-sectional design we cannot infer causality. For example, we cannot determine if staff training leads to higher levels of preparedness or if agencies with higher levels of preparedness are more likely to have staff training. Nevertheless, these data do provide a good snapshot of current preparedness among US ME/Cs, filling a gap in the literature. Second, self-reported (and therefore subjective) responses could lead to under- or overestimation of actual preparedness. However, if self-reporting bias does exist in the sample, our results are much more likely to represent an exaggerated degree of preparedness than might actually be the case, as ME/Cs with little or no preparedness might have been discouraged from completing the survey. Thus, we believe the preparedness gaps reported here represent the minimum of those found in the field, and that actual gaps may be even more dramatic.

Finally, because only a relatively small sample of the nation’s ME/Cs participated in this questionnaire, we therefore have the potential risk of response bias and lack of representativeness. Our sample therefore may not reflect the actual state of preparedness of the entire population of ME/Cs in the US. Our small sample and potential response bias of respondents may therefore lead to a lack of generalizability. However, our findings are similar to the very limited data presented in the 2012 and 2013 National Preparedness Reports and are also consistent with the perspectives offered by national leaders with broad knowledge of response capabilities in this sector [13, 51]. Our study also benefited from representation of every Federal Region. In the future, it would be helpful to conduct annual surveys on preparedness of this key sector. A more complete assessment of preparedness in this sector will likely be achieved through more robust recruitment and follow-up measures, larger samples, and prospective study designs using multivariable approaches in order to account for potential confounding variables. Actual experimental studies should also be conducted (preparedness training vs. wait list control) to identify evidence based training programs.

## Conclusions

Current climatological, meteorological, social and political trends point toward increased risk of disaster-related events and the possibility of MFI. We can mitigate the risk to some extent, but it is likely that MFI will continue. Therefore, it is imperative that we take the necessary steps to prepare for these as feasibly as possible. Even small preparedness steps can increase the effectiveness of mass fatality management, and this in turn can help support recovery of affected communities. Effective mass fatality management, which includes respectful, culturally sensitive handling of human remains, expeditious identification of the decedents, and rapid release of the remains to family members for final disposition, can help support the recovery and resiliency of survivors and the rehabilitation of communities. Effective MFI management shows both respect for the dead as well as compassion for the bereaved and is appropriately one of our nation’s priority goals for preparedness.

## Electronic supplementary material

Additional file 1: **NSF Mass Fatality Management Survey for MEC.** (PDF 412 KB)

Additional file 2: **MEC Code Book.** (DOCX 21 KB)

## Abbreviations

US: United States
ME/C: Medical Examiner or Coroner
MFI: Mass Fatality Incident
CFM: Complex Fatality Management
CBRNE: Chemical, biological, radiological, nuclear or explosive
NRF: National Response Framework
DHHS: Department of Health and Human Services
DMORT: Disaster Mortuary Operational Response Team
UCSF: University of California, San Francisco
CHR: Committee on Human Research
NIMS: National Incident Management System
NRP: National Response Plan
EMS: Emergency Support Function
FEMA: Federal Emergency Management Agency
CPG: Comprehensive Preparedness Guide
OR: Odds ratio
CI: Confidence interval
NTSB: National Transportation Safety Board
IAC & ME: International Association of Coroners & Medical Examiners
NAME: National Association of Medical Examiners.
