# Supplementary material for: Mass fatality preparedness among medical examiners/coroners in the United States: a cross-sectional study
Source: BMC Public Health. 2014 Dec 15;14:1275. doi: 10.1186/1471-2458-14-1275 (PMC4320632; doi:10.1186/1471-2458-14-1275)
Supplement: Supplementary file 1 — Additional file 1: NSF Mass Fatality Management Survey for MEC. (PDF 412 KB) [file 12889_2014_7476_MOESM1_ESM.pdf]

NSF Mass Fatality Management Survey for MEC

1. Introduction to the Study entitled: “Systems-Level Mass Fatality Preparedn...

Dear Colleague,

Your input on mass fatality management is very important to us. Thank you for taking a few minutes of your valuable time to complete this survey on your organization’s mass fatality incident preparedness. You can stop and restart the survey by simply using the **same PC/laptop and browser** when restarting.

To learn more about the study click here. The results from this survey will help inform emergency planning at the local and national level. Summary data will be made available on our website community at the completion of this study. Please note the survey closes on **March 31st, 2014**.

Please consider sharing the survey link with your colleagues who work in mass fatality incident preparedness and response.

All information is strictly confidential. Please feel free to contact Dr. Robyn Gershon (Primary Investigator) if you have any questions, comments or feedback.

Thank you for your time,

Robyn Gershon

Robyn R.M. Gershon, MT, MHS, DrPH
Professor
Department of Epidemiology and Biostatistics and
Philip R. Lee Institute for Health Policy Studies
School of Medicine
University of California, San Francisco
3333 California Street, Suite 280
San Francisco, CA 94118
robyn.gershon@ucsf.edu

2. Consent Form

UNIVERSITY OF CALIFORNIA, SAN FRANCISCO
CONSENT TO PARTICIPATE IN A RESEARCH STUDY

Study Title: Systems-Level Mass Fatality Preparedness

Why is this study being done?
This study is funded by the National Science Foundation. This study is designed to help researchers better understand how prepared organizations are for “mass fatality incidents.” The findings from this study will help organizations better prepare for mass fatality incidents.

We are particularly interested in learning more about organizations that are directly responsible for preparing for and responding to mass fatality incidents. These groups include: offices of medical examiners, coroners, local and state health departments, offices of emergency management, funeral homes, cemeteries, crematories, faith based organizations and DMORT teams. You are being asked to take part in this study because you are affiliated with one of these groups.

How many people will take part in this study?
About 10000 individuals who are involved with fatality management services and/or the death care industry will be asked to complete this survey. They will come from all 10 federal regions throughout the United States.

What are my rights if I take part in this study?
Taking part in this study is completely your choice. If you decide to take part, you will be asked to respond to questions about preparedness for mass fatality incidents. You can stop at any time, by simply closing the webpage. If you decide not to participate, there will be no penalty to you in any way.

How long will I be in this study?
This survey should take about 15-20 minutes to complete.

What risks are there from participation in this study?
The risks are that there could be a loss of security (e.g., data hackers), but we have taken precautions to keep the data secure.

What benefits are there from participation in this study?
There will be no direct benefit to you from participating in this study. However, the findings from the study questions may help your organization better prepare for a mass fatality incident.

Will information about me be kept private?
The survey and all of the study’s records are completely confidential. Only the Principal Investigator and the project coordinator will have the access to any identifying information. All survey data will be password protected and backed up daily to off-site locations. Organizations such as the UCSF Committee on Human Research and the National Science Foundation may look at and/or copy the study research records for various reasons. However, all of this study’s information is completely de-identified.

What are the costs of taking part in this study?
There are no costs for taking part in this study.

Will I be paid for taking part in this study?
There is no payment for taking part in this study.

Who can answer my questions about the study?
If you have any questions concerning this study or your participation in it, please contact the Principal Investigator, Dr. Robyn Gershon, by email at robyn.gershon@ucsf.edu. If you have any questions about your rights as a participant in this

study, you may discuss them with the University of California, San Francisco Committee on Human Research at 415-476-1814 for further information.

Signature Section

By clicking "Next," I am confirming that:

1) I am 18 years of age or older. 2) I have never taken this survey before. 3) I understand that my participation in this study is voluntary and that I can leave the survey at any time without prejudice by navigating away from the survey webpage. 4) I agree to enter this research study and I am providing my electronic signature on this consent form.

NSF Mass Fatality Management Survey for MEC

3. Definition & Notice

Mass Fatality Incident Definition: Any situation where <u>more</u> deaths occur in a point in time than can be typically handled by your office.

4. Survey Questions

1. Which of the following agencies/offices/organizations do you primarily represent?

- ◯ Medical Examiner
- ◯ Coroner
- ◯ County Sheriff
- ◯ Justice of the Peace
- ◯ Other (please specify)

2. How did you receive this survey? Please check all that apply.

- ☐ National Association of Medical Examiners (NAME)
- ☐ The International Association of Coroners and Medical Examiners (IAC&ME)
- ☐ National Sheriffs' Association
- ☐ Other (please specify)

3. What is your agency/office/organization's zip code?

Please input your zip code

4. What is the approximate size of the population that your agency/office/organization serves?

5. Your State

6. Approximately, how many full time employees are in your agency/office/organization?

Number of full time employees

7. Given your resources, approximately how many fatalities (within 48 hours) would exceed your capacity to respond?

8. In the last 5 years, has your jurisdiction experienced a mass fatality incident?

- ◯ Yes
- ◯ No
- ◯ Don't know

9. During a mass fatality incident, does your agency/office/organization have a seat at your jurisdiction's Emergency Operations Center?

- ◯ Yes
- ◯ No
- ◯ Don't know

10. During a mass fatality incident, does your agency/office/organization have a defined position?

- ◯ Yes
- ◯ No
- ◯ Don't know

11. Does your agency/office/organization have a <u>written Mass Fatality Plan</u>?

- ◯ Yes
- ◯ No
- ◯ Don't know

6. Survey Questions

12. If yes, how often is this updated?

◯ Every year
◯ Every two years
◯ Every five years
◯ Never; it has not been updated
◯ Don't know
◯ Other (please specify)

13. If yes, is your plan compliant with the National Incident Management System (NIMS)?

◯ Yes
◯ No
◯ Don't know

14. If yes, is your plan compliant with FEMA Comprehensive Preparedness Guide (CPG) 101?

◯ Yes
◯ No
◯ Don't know

15. Does your plan include interoperability and mutual aide agreements to allow for sharing resources (e.g., personnel, equipment, materials, and other associated services) with your jurisdictional partners?

- ◯ Yes
- ◯ No
- ◯ Don't know

16. Does your plan include the following? Please check all that apply.

- ☐ Applicability and Scope
- ☐ Assumptions
- ☐ Authorities (legal and statutory authorities)
- ☐ Funding Reimbursement
- ☐ Concept of Operations
- ☐ Continuity of Operations Plan
- ☐ Credentialing, Managing and Documenting Disaster Personnel, including Volunteers
- ☐ Incident Notification and Plan Activation
- ☐ Command and Control
- ☐ Job Action Sheets for the Various Positions in the Plan
- ☐ Human Remains Recovery
- ☐ Morgue Services
- ☐ Family Assistance
- ☐ Staff Respite Area
- ☐ Vital Records System
- ☐ Mass Fatality Information Systems
- ☐ Religious/cultural Considerations (e.g., Disaster Emotional & Spiritual Care, Chaplaincy)
- ☐ Security and preservation of the remains
- ☐ Security and preservation of the disaster site
- ☐ Our plan does not have any of these elements
- ☐ Other (please specify)

8. Survey Questions

17. Do any of your jurisdictional partners have a Mass Fatality Plan?

- ◯ Yes
- ◯ No
- ◯ Don't Know

18. Has your agency/office/organization signed off on the mass fatality plans of your jurisdictional partners?

- ◯ Yes
- ◯ No
- ◯ Don't know

9. Survey Questions

19. If yes, please indicate which partners' plans you have signed off on: (Please check all that apply.)

- ☐ Local Emergency Management Agency
- ☐ Local Department of Health
- ☐ Law Enforcement
- ☐ Fire/Rescue/Hazardous Materials
- ☐ Emergency Medical Services
- ☐ Local Health Care Organizations (e.g., hospitals)
- ☐ Other (please specify)

20. Does your agency/office/organization have written policies regarding <u>public communications</u> (e.g., public announcements or instructions) during a mass fatality incident?

- ◯ Yes
- ◯ No
- ◯ Don't know

21. Does your agency/office/organization have written policies regarding use of <u>social media</u> during a mass fatality incident?

- ◯ Yes
- ◯ No
- ◯ Don't know

22. Does your agency/office/organization have or have access to a <u>written mental health/spiritual counseling plan</u> to manage the traumatic reactions of your employees and/or volunteers during and after the response to a mass fatality incident?

- ◯ Yes
- ◯ No
- ◯ Don't know

10. Survey Questions

23. Does your agency/office/organization have the following OPERATIONAL CAPABILITIES (i.e., on your own - without your response partner's help) with respect to managing a mass fatality incident? Please check all that apply.

- ☐ Command and Control for Fatality Management
- ☐ Security and Preservation of Disaster Site
- ☐ Incident Characterizations
- ☐ Decedent Manifest
- ☐ Public Messaging
- ☐ Communication via Social Media
- ☐ Missing Persons Call Centers
- ☐ Information Technology/Tracking
- ☐ Joint Agency Death Investigation
- ☐ Decedent Recovery
- ☐ Tracking system (i.e., Victim Identification Program) for Recovered Remains
- ☐ Antemortem Data Collection
- ☐ Security and Preservation of Human Remains
- ☐ Postmortem Examination/Morgue Operations
- ☐ Morgue Operations for Contaminated (hazardous materials) Human Remains
- ☐ Transport of Remains
- ☐ Refrigerated Storage of Remains
- ☐ Temporary Interment
- ☐ Caring for or Interring Human Remains in accordance to the Religious Ritual or Requirements of most Faith Traditions
- ☐ Decedent Release/Final Disposition
- ☐ Long Term Family Management/Memorial
- ☐ Our agency/office/organization do not have any of these operational capabilities
- ☐ Other (please specify)

24. If a large-scale mass fatality incident (e.g., pandemic, earthquake) occurred, which additional resources would you require in order to respond? Please check all that apply.

- ☐ Additional refrigerated morgue storage space
- ☐ Additional qualified personnel who are available to report to work (professional staff, investigative staff, and/or transport staff)
- ☐ Additional equipment and supplies (e.g., body bags, personal effects bags, or vehicles needed for transport)
- ☐ Additional temporary burial site
- ☐ Other (please specify)

25. Which of the following agencies/organizations does your agency/office/organization have pre-existing relationships with (either directly or through Office of Emergency Management) in order to obtain these additional resources? Please check all that apply.

- ☐ Disaster Management Vendors/contractors
- ☐ State Emergency Management Agency/Civil Defense
- ☐ Local Emergency Management Agency/Civil Defense
- ☐ Other nearby Office of Medical Examiner
- ☐ Other nearby Coroner/Sheriff's Office/Justice of the Peace
- ☐ Local first response organizations (e.g., fire, EMS, police)
- ☐ Local/State Departments of Health
- ☐ Local health care organizations (e.g., hospitals)
- ☐ Local funeral homes, cemeteries, crematories
- ☐ Voluntary organizations (e.g., Red Cross, Neighborhood Emergency Response Team [NERT], Community Emergency Response Teams [CERT])
- ☐ Faith-based organizations (e.g., Disaster Chaplaincy, Disaster Emotional & Spiritual Care providers)
- ☐ Federal assets (e.g., DMORT teams, National Guard, Department of Defense, Department of Energy, etc.)
- ☐ Other (please specify)

26. Which organizations/agencies would your agency/office/organization expect to provide resources to during a mass fatality incident? Please check all that apply.

- [ ] State Emergency Management Agency/Civil Defense
- [ ] Local Emergency Management Agency/Civil Defense
- [ ] Other nearby Office of Medical Examiner
- [ ] Other nearby Coroner/ Sheriff's Office/Justice of the Peace
- [ ] Local first response organizations (e.g., fire, EMS, police)
- [ ] Local/State Departments of Health
- [ ] Local health care organizations (e.g., hospitals)
- [ ] Local funeral homes, cemeteries, crematories
- [ ] Voluntary organizations (e.g., Red Cross, NERT,CERT)
- [ ] Faith-based organizations
- [ ] Federal assets (e.g., DMORT teams, National Guard, Department of Defense, Department of Energy, etc.)
- [ ] Other (please specify)

27. Has your agency/office/organization provided training to its staff on your mass fatality plan?

- ○ Yes
- ○ No
- ○ Don't know
- ○ We do NOT have a mass fatality plan

28. Have your agency/office/organization's staff been trained/certified on mass fatality incidents that involve CBRNE (Chemical, Biological, Radiological, Nuclear and high-yield Explosives)?

- ○ Yes
- ○ No
- ○ Don't know

29. Has your agency/office/organization participated in city-wide, county-wide and/or state-wide drills/exercises on mass fatality incidents?

- ◯ Yes
- ◯ No
- ◯ Don't know

30. Has your agency/office/organization created a staff roster to determine the <u>number of staff</u> that would likely to be able to report for duty during a mass fatality incident?

- ◯ Yes
- ◯ No
- ◯ Don't know

31. In *your professional opinion*, what proportion of your agency/office/organization's staff would likely to be WILLING (willingness refers to being favorably inclined) to report in their roles during a mass fatality incident?

32. In *your professional opinion*, what proportion of your agency/office/organization's staff would likely to be WILLING to report in their roles during a mass fatality incident that involved CBRNE (Chemical, Biological, Radiological, Nuclear and high- yield Explosives) contaminants?

33. In *your professional opinion*, what proportion of your staff would likely to be ABLE (available, if not directly impacted by the event) to report in their roles during a mass fatality incident?

34. In *your professional opinion*, what proportion of your staff would likely to be ABLE to report in their roles during a mass fatality incident involved CBRNE (Chemical, Biological, Radiological, Nuclear and high-yield Explosives) contaminants?

35. In *your professional opinion*, what proportion of your staff have pre-event plans in place that would ensure their availability to report in their roles during a mass fatality incident?

36. In *your professional opinion*, how would you rate the overall preparedness of your agency/office/organization?

37. In *your professional opinion*, how would you rate the overall preparedness of the jurisdiction (e.g., city, county, state) that your agency/office/organization serves?

38. What do you think your agency/office/organization needs in order to be <u>better prepared</u>? Please check all that apply.

- [ ] More funding for mass fatality planning
- [ ] More mass fatality planning activities
- [ ] A written mass fatality plan
- [ ] More signed interagency agreements
- [ ] More drills with other response partners
- [ ] More training of staff
- [ ] Greater surge capacity (identification of additional staff, supplies, space)
- [ ] I don’t think my agency/office/organization needs anything else to be better prepared
- [ ] Other (please specify)
