# Supplementary material for: Mass fatality preparedness among medical examiners/coroners in the United States: a cross-sectional study
Source: BMC Public Health. 2014 Dec 15;14:1275. doi: 10.1186/1471-2458-14-1275 (PMC4320632; doi:10.1186/1471-2458-14-1275)
Supplement: Supplementary file 2 — Additional file 2: MEC Code Book. (DOCX 21 KB) [file 12889_2014_7476_MOESM2_ESM.docx]

**MEC Code Book**

| Question Item # | Variable | Recoded Responses | Original Coding |
| --- | --- | --- | --- |
| Demographic Variables | | |  |
| Q1 | Workplace category  q1wcate | 1= Medical Examiner  2= Coroner  na= Other or missing | 1= Medical Examiner  2= Coroner  3= Other  88= Missing |
| Q6 | Approx. full time employee  q6ftemp | 0= 6 or less  1= 7 or more  na= missing | 1= Below 6  2= 7-25  3= 26-50  4= 51-100  5= Above 100  88= Missing |
| Q7 | Approx. number of fatalities exceed capacity  q7fcap | 0= 24 or less (1)  1= 25 or more (2-5)  na= Don’t know or missing | 1= Below 25  2= 25 – 50  3= 51 – 75  4= 76 – 100  5= Above 100  99= Don’t know  88= Missing |
| Q8 | Experience of MFI in the past 5 years  q8mfi5yrs | 0=No  1=Yes na=Don’t know or missing | 1= Yes  0= No  99= Don’t know  88= Missing |
| Q16 | Total score of Q16 (elements in mass fatality plan)  q16total | 0-19 (min=0, max=19) | 0= No  1= Yes |
| Q23 | Total score of Q23 (operational capabilities)  q23total | 0-21 (min=0, max=21) | 0= No  1= Yes |
| Q16+Q23 | Total score of Q16 + Q23  q1623total | 0-40 (min=0, max=40) | N/A |
| Q27 | Trainings to staff on MFP  q27trainmfp | 0= No or not have a plan (0, 2)  1= Yes  na= Don’t know or missing | 1= Yes  0= No  2= We do NOT have a mass fatality plan  99= Don’t know  88= Missing |
| Q28 | Training on CBRNE  q28traincbrne | 0=No  1=Yes  na=Don’t know or missing | 1= Yes  0= No  99= Don’t know  88= Missing |
| Q29 | Drills participation  q29drills | 0= No  1= Yes  na= Don’t know or missing | 1= Yes  0= No  99= Don’t know  88= Missing |
| Q30 | Staff roster of # of staff able to report  q30sroster | 0= No  1= Yes  na= Don’t know or missing | 1= Yes  0= No  99= Don’t know  88= Missing |
| Q31 | Willingness to report in regular MFI  q31wmfi | 0= 70% or less (1-8)  1= 80% or more (9-11)  na= Don’t know or missing | 1= 0%  2= 10%  3= 20%  4= 30%  5= 40%  6= 50%  7= 60%  8= 70%  9= 80%  10= 90%  11= 100%  99= Don’t know  88= Missing |
| Q32 | Willingness to report in CBRNE involved MFI  q32wcbrne | 0= 70% or less (1-8)  1= 80% or more (9-11)  na= Don’t know or missing | 1= 0%  2= 10%  3= 20%  4= 30%  5= 40%  6= 50%  7= 60%  8= 70%  9= 80%  10= 90%  11= 100%  99= Don’t know  88= Missing |
| Q33 | Able to report in regular MFI  q33amfi | 0= 70% or less (1-8)  1= 80% or more (9-11)  na= Don’t know or missing | 1= 0%  2= 10%  3= 20%  4= 30%  5= 40%  6= 50%  7= 60%  8= 70%  9= 80%  10= 90%  11= 100%  99= Don’t know  88= Missing |
| Q34 | Able to report in CBRNE involved MFI  q34acbrne | 0= 70% or less (1-8)  1= 80% or more (9-11)  na= Don’t know or missing | 1= 0%  2= 10%  3= 20%  4= 30%  5= 40%  6= 50%  7= 60%  8= 70%  9= 80%  10= 90%  11= 100%  99= Don’t know  88= Missing |
| Q35 | Proportion of staff have pre-event plans  q35preeventp | 0= 70% or less (1-8)  1= 80% or more (9-11)  na= Don’t know or missing | 1= 0%  2= 10%  3= 20%  4= 30%  5= 40%  6= 50%  7= 60%  8= 70%  9= 80%  10= 90%  11= 100%  99= Don’t know  88= Missing |
| Q36 | Overall preparedness of workplace  q36wprep | 0= 3 or less (1-3)  1= 4 or more (4-5)  na= Don’t know or missing | 1= 1- not at all prepared  2= 2  3= 3  4= 4  5= 5 – completely prepared  99= Don’t know  88= Missing |
| Q37 | Overall preparedness of the jurisdiction  q37jprep | 0= 3 or less (1-3)  1= 4 or more (4-5)  na= Don’t know or missing | 1= 1- not at all prepared  2= 2  3= 3  4= 4  5= 5 – completely prepared  99= Don’t know  88= Missing |
| Q39 | FEMA Region  q39fema | 2-digit Number (1-10) | Code per questionnaire  88=Missing |
| Q40 | Urban or Rural Area  q40ua | 0= Rural Area  1= Urban Area  99= Don’t know  88= Missing |  |
